# Efficacy of *Dodonaea viscosa* Extract and Its Chitosan-Based Nanoparticle Formulation on the Growth of *Fusarium* Strains and the Production of Deoxynivalenol and Moniliformin in Stored Wheat

**DOI:** 10.3390/toxins17110551

**Published:** 2025-11-05

**Authors:** Hussein Ali Salman Alsahho, Sumer Horuz, Kevser Karaman

**Affiliations:** 1 Graduate School of Natural and Applied Sciences, Erciyes University, 38280 Kayseri, Türkiye; 2 Iraqi Ministry of Trade-Grain Board of Iraq, Bab-Al Moazzam, Baghdad 329, Iraq; 3 Faculty of Agriculture, Department of Plant Protection, Erciyes University, 38280 Kayseri, Türkiye; 4 Faculty of Agriculture, Department of Agricultural Biotechnology, Erciyes University, 38280 Kayseri, Türkiye; kevserkaraman@erciyes.edu.tr

**Keywords:** *Dodonaea viscosa*, deoxynivalenol, moniliformin, *Fusarium*, nanotechnology, stored wheat

## Abstract

*Fusarium* is considered one of the most important fungi that attack plants and cause serious diseases resulting in huge losses to crops, especially wheat. Fungicides have been used to control it, but they have drawbacks, including residues and toxicity to mammals, which encouraged researchers to find alternatives to these methods and materials. This study was conducted to find natural alternatives to the chemicals used as fungicides. The *Dodonaea viscosa* plant extract was evaluated as an extract (DVE) and nanoparticles (chitosan NPs loaded with DVE) to inhibit the growth of *Fusarium* spp. strains and production ability of Deoxynivalenol (DON) and Moniliformin (MON) mycotoxins. The wheat samples were taken from storage in eighteen different governorates in Iraq. *Fusarium* spp. strains were detected phenotypically, and seven strains were identified by using the polymerase chain reaction technique (PCR) as *F. oxysporum*, *F. pseudograminearum* and *F. chlamydosporum*. DVE effectively inhibited the growth of *Fusarium* spp. strains at three different concentrations (0.5, 1.0, and 1.5%) on PDA. The highest percentage was 68.94% for *F. oxysporum* strain 5, and the lowest percentage was 22.58% for *F. pseudograminearum* strain 6 at a concentration of 1.5%. However, applying chitosan NPs loaded with DVE at a concentration of 0.75% effectively increased the inhibition rate. The treatment of chitosan NPs loaded with DVE played a role in inhibiting the percentage of mycotoxins produced. The highest percentage of inhibition of the DON toxin was recorded as 73.75% in *Fusarium pseudograminearum* strain 2, and the highest percentage of inhibition of the production of the (MON) toxin was 73.62% in isolate *Fusarium chlamydosporum* strain 8. Overall, this study highlights for the first time the potential of *Dodonaea viscosa* nano-formulation to suppress both fungal growth and mycotoxin biosynthesis, providing a sustainable and safe strategy for protecting stored grains.

## 1. Introduction

Certain fungi produce toxic secondary metabolites, namely mycotoxins, in cereals, nuts, and fruits, that affect humans and animals when grains are used in the human diet and animal feed [1,2]. One of the concerns in wheat production is the possible accumulation of mycotoxins, both as a commercial risk and as a health risk. Among the mycotoxigenic fungi, *Fusarium* spp. are of particular importance, as they can infect grains both pre- and post-harvest, and the infection may persist during storage, resulting in quality deterioration and grain loss [3]. To date, more than 400 mycotoxins have been identified [4], with deoxynivalenol (DON), nivalenol, zearalenone, T-2 toxin, and fumonisin B1 recognized as the most prevalent and hazardous compounds [5]. DON, also referred to as *vomitoxin*, is a type B trichothecene mycotoxin frequently detected in wheat and commonly produced by *Fusarium pseudograminearum* [6]. Moniliformin (MON) is another important mycotoxin produced by various *Fusarium* species, and, to date, approximately 40 species have been reported as MON producers [7,8].

The use of extracts and natural materials as an alternative to chemical pesticides is the goal of many researchers. Due to the adverse effects of synthetic pesticides on the environment, humans, and animals, the search for safer alternative pest control strategies has become imperative. Natural compounds and extracts of plants have been analyzed in many studies for their antifungal activities and the identification of the ergosterol synthesis inhibition mechanism [9]. *Dodonaea viscosa* also known as the broadleaf hopbush, is a flowering plant belonging to the *Sapindaceae* family. It has been used in the treatment of burns, teeth, skin infections, stomach pain, etc., and has an inhibitory effect against microbes and insects as an effective pesticide, and it has also antioxidant and antiparasitic activity [10]. A flavone compound 5,6,8-trihydroxy-7,4′ dimethoxy flavone was isolated from the crude extract of *Dodonaea viscosa* var. *angustifolia* Jacq has demonstrated antifungal activity by inhibiting ergosterol synthesis in *C. albicans* with an MIC value of 0.39 mg/mL and MFC value of 1.56 mg/mL [11]. The antimicrobial (particularly antibacterial) activity of *Dodonaea viscosa* has been clearly confirmed in the study. The methanol extract alone exhibits a significant effect against both Gram-positive (particularly *S. aureus* and *B. subtilis*) and Gram-negative (*E. coli*, *K. pneumoniae*) bacteria. MIC values have decreased to levels as low as 500–1000 µg/mL in some pathogens [12]. In the study conducted by Khurram et al. (2009), the plant’s ethanolic extract and various solvent fractions (particularly ethyl acetate and n-butanol) formed strong inhibition zones and showed a marked effect on pathogens such as *S. aureus*, *M. luteus*, *E. coli* and *P. aeruginosa* [13]. In order to enhance the stability, solubility, and bioefficacy of plant extracts, nanotechnology-based delivery systems have recently gained significant attention—a strategy also adopted in the present study.

Nanotechnology can be defined as the process of producing materials with one or more dimensions on a scale of 100 nm or less [14]. The extracts could be converted into nanoparticles by binding them to nano chitosan, where chitosan has been used in agricultural applications due to its important properties such as biodegradability and environmental friendliness when used in fertilizers and seed coatings [15]. The effectiveness of chitosan nanoparticles loaded with *Syzygium aromaticum* extract against *Klebsiella pneumoniae* has been demonstrated in a previous study [16]. In another study, there was a clear effect of the produced CH@CuO nanoparticles on cultivated tomato plants infected with isolates of *F. oxysporum* [17]. In addition, they are used as nanomaterials due to the combination of fungicide or chemicals with chitosan through ionic or covalent bonds or encapsulating these materials with chitosan matrices due to their homogeneous and stable encapsulation property [18].

Therefore, the main objective of this study was to evaluate the antifungal efficiency of *Dodonaea viscosa* extract and its nano-formulation against *Fusarium* species isolated from stored wheat, with particular emphasis on their potential to inhibit fungal growth and the biosynthesis of the mycotoxins deoxynivalenol (DON) and moniliformin (MON). In addition, the study aimed to compare the effectiveness of the conventional extract and its nano-chitosan-loaded form to highlight the role of nanotechnology in enhancing the bioactivity, stability, and efficacy of plant-derived antifungal agents.

## 2. Results and Discussion

### 2.1. Wheat Sampling and Identification of Fusarium *spp.* Strains

A total of 51 stored wheat samples were collected from eighteen governorates in Iraq, from which seven *Fusarium* strains were successfully isolated, each originating from a different governorate (Nineveh, Maysan, Dohuk, Anbar, Ta’mim, Salah al-Din, and Basra). These strains belonged to three *Fusarium* species: *Fusarium oxysporum*, *F. pseudograminearum*, and *F. chlamydosporum*.

On PDA medium, the colonies exhibited soft, pale, cottony mycelial growth. Strains 4 and 5 were identified as *F. oxysporum*, showing colony colors ranging from white to pale purple. Strains 2, 3, 6, and 7 were classified as *F. pseudograminearum*, with colonies displaying white to cream pigmentation. Strain 8 was identified as *F. chlamydosporum*, characterized by a light pink to dark red colony color.

The genetic identity of all strains was confirmed by polymerase chain reaction (PCR) analysis, as presented in Table 1.

**Figure 1:**
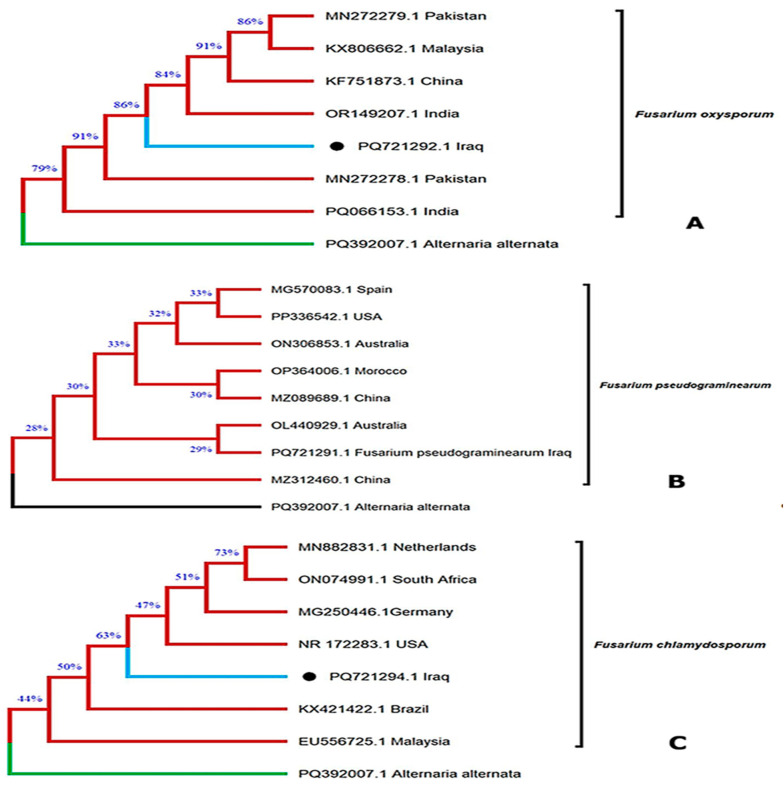
Phylogenetic tree was constructed for three isolates belonging to three *Fusarium* species isolated from wheat stored in Iraq. (**A**): *F. oxysporum *(PQ721292.1 Iraq); (**B**): *F. pseudograminearum *(PQ721291.1 Iraq); (**C**): *F. chlamydosporum *(PQ721294.1 Iraq).

Three *Fusarium* strains representing the species previously identified morphologically were further confirmed at the molecular level and deposited in the GenBank database. Their corresponding accession numbers are shown in Figure 1: *F. oxysporum* (PQ721292.1, Baghdad, Iraq; Figure 1A), *F. pseudograminearum* (PQ721291.1, Baghdad, Iraq; Figure 1B), and *F. chlamydosporum* (PQ721294.1, Baghdad, Iraq; Figure 1C). The phylogenetic clustering of these isolates with reference strains from different geographical regions validates their taxonomic identity and demonstrates their genetic relatedness at a global scale. This molecular confirmation not only supports the accuracy of strain identification but also contributes to the enrichment of publicly available *Fusarium* genomic data from Iraq.

The findings of this study are in agreement with previous reports documenting the occurrence of *Fusarium pseudograminearum*, *F. graminearum*, *F. boothii*, and *F. culmorum* in infected wheat samples across Iraq [19]. Similarly, during the 2020/2021 agricultural season, *F. pseudograminearum* was isolated from wheat samples collected from fields in the Basra, Maysan, and Dhi Qar governorates. The present detection of *F. pseudograminearum* is therefore consistent with earlier observations from Basra, where multiple *Fusarium* species, including *F. pseudograminearum*, were reported in both wheat and barley crops [20]. Furthermore, *F. chlamydosporum* was previously reported for the first time in wheat in Iraq in that study, and its occurrence is reaffirmed by the current research.

Importantly, *F. pseudograminearum* and *F. chlamydosporum* are recognized as major causal agents of *Fusarium* crown rot (FCR), *Fusarium* head blight (FHB), and *Fusarium* root rot (FRR), posing a serious threat to wheat production systems in Iraq [21].

### 2.2. Testing the Effect of DVE on the Growth of Fusarium *spp.* Strains

The inhibitory effect of DVE on the tested *Fusarium* strains increased progressively with rising extract concentration (Table 2). At 0.5% extract concentration, the highest growth inhibition was observed in *F. oxysporum* strain 5 (63.37%), whereas the lowest was recorded in *F. pseudograminearum* strain 6 (15.16%). Increasing the extract concentration to 1.0% further enhanced the inhibitory effect, with *F. oxysporum* strain 5 again showing the strongest response (67.13%), while *F. pseudograminearum* strain 6 remained the least affected (20.64%). The maximum inhibition levels were recorded at 1.5% DVE, reaching 68.94% in *F. oxysporum* strain 5, compared to only 22.58% inhibition in *F. pseudograminearum* strain 6.

Analysis of variance confirmed that the effect of DVE was statistically significant across all strains and concentrations. Post hoc LSD analysis further revealed that the inhibition at 1.5% was significantly higher than at 0.5% for all strains, while the differences between adjacent concentrations (0.5% vs. 1.0% and 1.0% vs. 1.5%) were comparatively less pronounced.

These findings align with earlier reports indicating potent antifungal activity of DVE against pathogenic fungi such as *Aspergillus niger* and *A. flavus*. The variability in strain sensitivity may be attributed to differences in the phytochemical profile of the extract, which depends largely on the solvent used for extraction. Previous studies similarly reported that ethanolic extracts of *D. viscosa* displayed variable inhibitory effects across fungal species [22].

### 2.3. Identified Major Components of DVE Using GC-MS System

A total of 27 compounds were detected in the DVE based on gas chromatography–mass spectrometry (GC–MS) analysis (Table 3). The major chemical classes identified were glycosides, saponins, and alkaloids. The most abundant compound was 4-Cyclohexyl-1-(furan-2-ylmethyl)-4H,5H,7H-pyrazolo [3,4-b]pyridin-6-one, followed by 4′,5,7-trihydroxy-3,6-dimethoxyflavone, 6-methoxy-2-[2-(imidazol-1-yl)ethoxy]-8-nitroquinoline, and 3-heptanol (TMS derivative). *Dodonaea viscosa* is a member of the *Sapindaceae* family and has been reported to contain antimicrobial, antitoxic, and cytotoxic bioactive compounds [22]. It also contains catechol, a phenolic compound known for its inhibitory effects against certain fungi and bacteria, including *Fusarium oxysporum* and *Penicillium italicum* [23]. In addition, neophytadiene, a terpenoid constituent of the leaf extract, has been documented as a strong antioxidant and an active antimicrobial agent against fungal and microbial infections [24].

Omosa et al. (2014) further reported the isolation of flavonoids such as 5-hydroxy-3,4′,7-trimethoxyflavone, santin, and pinocembrin, as well as diterpenoids including dodonic acid and hautriwaic acid from the leaf surface exudates of *D. angustifolia*. These phytochemical classes exhibit strong chemical similarity to the major constituents identified in the present study. Therefore, the current findings strongly confirm that *Dodonaea viscosa* possesses a phytochemical profile predominantly characterized by flavonoids and diterpenoids [25].

### 2.4. Testing the Effect of Chitosan NPs Loaded with DVE on Fusarium *spp.* Strains

The antifungal activity of Chitosan NPs loaded with DVE was evaluated at a concentration of 0.75%, which corresponds to the average concentration used for the alcoholic extract in the standard formulation. As shown in Table 4, the nanoparticles exhibited a clear inhibitory effect against all *Fusarium* strains tested. The highest inhibition rate (70.58%) was recorded against *F. pseudograminearum* strain 7, whereas the lowest inhibition rate (61.17%) was observed in *F. chlamydosporum* strain 8. Statistical analysis confirmed that the treatment exerted a significant inhibitory effect (LSD = 6.429 *), with *F. pseudograminearum* strain 7 being the most susceptible strain.

Encapsulation of plant-derived extracts into nanocarriers is known to overcome limitations such as poor stability, limited bioavailability, and degradation of active compounds during delivery. In this study, nano-chitosan was used as a protective and controlled-release carrier for DVE, enhancing its antifungal efficiency—a finding consistent with previous reports on chitosan-based phytonanocarriers [26].

### 2.5. Conformational and Morphological Analysis of Chitosan NPs Loaded with DVE

**Figure 2:**
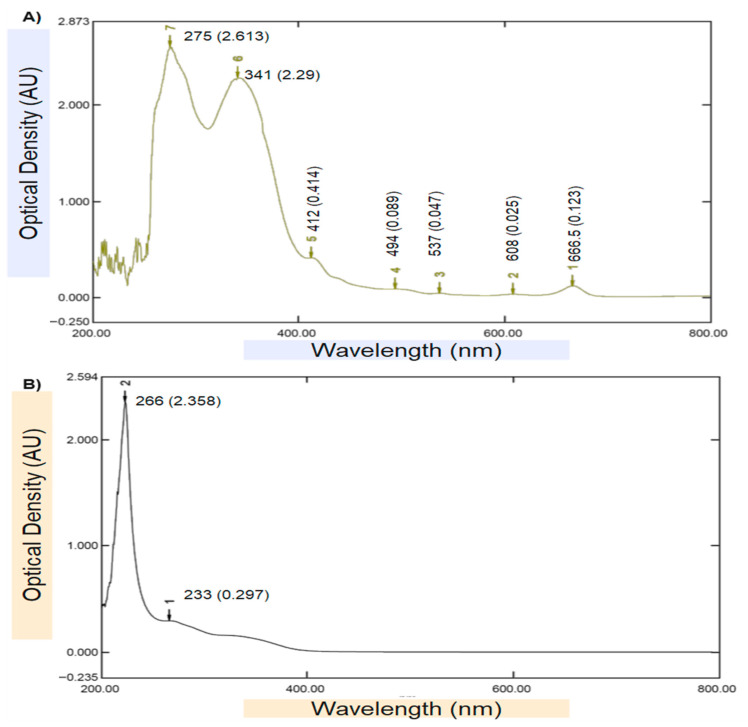
UV–Visible spectrophotometer test: (**A**). DVE, (**B**). Chitosan NPs loaded with DVE.

Ultraviolet (UV–Vis) spectrophotometer was used as an initial confirmatory technique to verify the biosynthesis of chitosan NPs loaded with DVE. The UV–Vis spectrum of the DVE (Figure 2A) exhibited two characteristic absorption bands at 266 nm (OD ≈ 2.358) and 233 nm (OD ≈ 0.297), which are typical for aromatic phenolic and flavonoid compounds. In contrast, the spectrum of the DVE-loaded chitosan nanoparticles (Figure 2B) revealed a distinct bathochromic (red) shift in the main peak toward 275 nm (OD ≈ 2.613), accompanied by a secondary absorption band at 341 nm, indicating strong intermolecular interactions between DVE phytochemicals and the protonated amino groups of chitosan. This wavelength shift—along with the notable increase in absorbance intensity—confirms the successful encapsulation and molecular stabilization of DVE within the chitosan.

Furthermore, the broad secondary absorbance region extended across the 400–700 nm range in the nanoparticle spectrum suggests enhanced light scattering due to nanoscale colloidal behavior and surface roughness, which is typical of plant-derived biopolymeric nanocarriers. The absence of additional degradation peaks suggests that no photolytic breakdown occurred during nanoparticle synthesis. Overall, the observed spectral changes strongly affirm the formation of chitosan NPs loaded with DVE and are consistent with previous findings for plant-extract-based biopolymeric nanocarriers [27,28].

**Figure 3:**
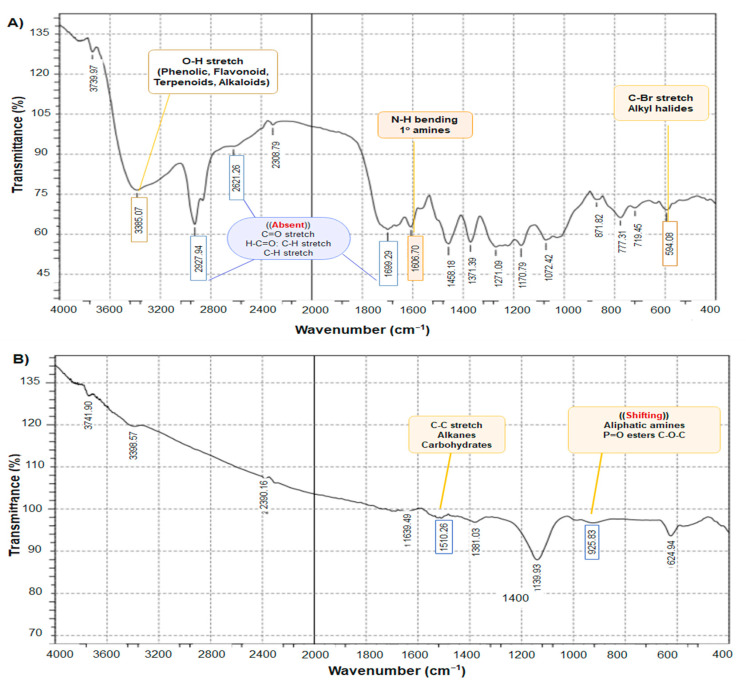
FTIR spectra (**A**)—DVE, (**B**)—chitosan NPs loaded with DVE.

Figure 3 exhibits the FTIR spectra of the DVE and chitosan NPs loaded with DVE and shows distinct peaks characteristic of various biomolecules, including phenol and alcohol (3610–3640 cm^−1^). A key observation is the difference in intensity between the amide I peak (1699.29) cm^−1^ in DVE spectrum (Figure 3A) and the corresponding peak (1639.94) cm^−1^ in the DVE-loaded chitosan NPs spectrum (Figure 3B). The higher intensity in the nanoparticle’s spectrum suggests a higher concentration of amides associated with the nanoparticles. This finding could be attributed to the attachment of many biomolecules during the synthesis process, potentially acting as a stabilizing agent. On the other hand, the absence of some peaks in chitosan NPs loaded with DVE (1150–1300, 1685–1710, 2850–3000 cm^−1^) referred to the attachment between DVE, and chitosan molecules and our results agreed with the result of the previous study [29]. When the C-H stretch was seen at 2927 cm^−1^, it provided structural information on chitosan.

By comparing the DVE/chitosan NPs loaded with DVE, it was noticed that the absence of many peaks due to the cross-linking of TPP may be attributed to the fact that the large molecules were fragmented into smaller units. The interaction of chitosan with TPP was the reason for the increase in the total surface area as shown in Figure 3B, indicating that the repositioned chemical groups facilitated the efficient loading of the extract into the chitosan/TPP matrix and the formation of nano-encapsulation of the extract. As additional supporting evidence, the shifted peaks are consistent with the formation of a new complex. The modifications in the functional groups of bioactive molecules suggest a relationship with the synthesis of DVE/chitosan NPs [30].

**Figure 4:**
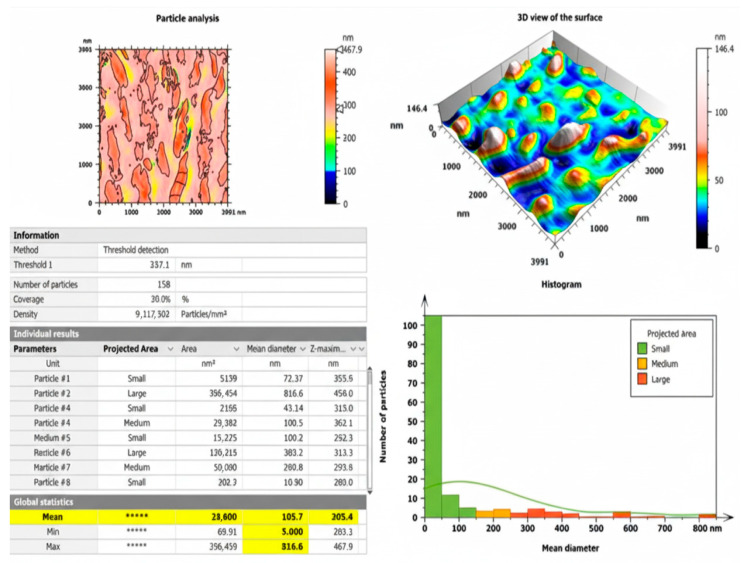
AFM surface morphology and particle size distribution of chitosan NPs loaded with DVE.

The AFM analysis of the biosynthesized chitosan NPs loaded with DVE is presented in Figure 4. A total of 158 individual nanoparticles were characterized, with a particle density of 9.92 × 10^6^ particles/mm^2^. The particles exhibited a high degree of morphological heterogeneity, showing predominantly elongated and plate-like structures with occasional sharp edges and clustered aggregates.

According to the statistical output, the mean particle diameter was 105.7 nm, which is consistent with the expected nanoscale range. The surface topography displayed a relatively rough and uneven texture, as confirmed by the 3D scan, which enhances the available surface area for potential interactions with bioactive molecules such as plant-derived compounds. The mean Z-height of the particles was 205.4 nm, indicating pronounced vertical protrusions rather than a flat morphology.

The observed nanoparticle aggregation could partially be attributed to sample handling and drying effects during AFM preparation, which is a common observation in chitosan-based nanostructures. These findings are in agreement with [16], who reported similar AFM features for clove-extract-loaded chitosan nanoparticles, showing non-spherical particles and moderate aggregation behavior due to intermolecular interactions between chitosan and phytochemical constituents.

**Figure 5:**
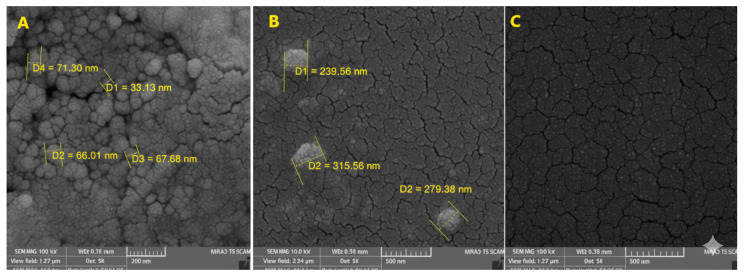
SEM micrographs of chitosan NPs loaded with DVE: (**A**) primary nanoparticles (100,000×), (**B**) secondary agglomerates after drying (50,000×), and (**C**) irregular surface morphology with apparent porosity (30,000×).

Figure 5A shows that the spherical nanoparticles with diameters of approximately 57–86 nm indicate the successful ionic gelation reaction with TPP. This morphological feature is commonly observed in chitosan-based nanoparticle systems and represents the early-stage state of nanoparticle aggregation following fabrication (Figure 5B). In the 30kX image (Figure 5C), the surface appears more complex and interlaced, where high-density regions composed of tightly packed nanoparticles are interspersed with low-density areas exhibiting pore- or void-like structures. These structural differences may be attributed to the non-homogeneous dispersion of the plant extract within the chitosan matrix or rapid solvent evaporation, which can lead to surface shrinkage. Overall, the majority of the nanoparticles exhibited a spherical morphology with a relatively uniform size distribution ranging between 38 and 79 nm.

### 2.6. Effect of DVE and Chitosan NPs Loaded with DVE on the Production of DON and MON Mycotoxins

The samples were analyzed using an HPLC system, and the corresponding chromatograms are provided in Supplementary File S1. Statistical analysis revealed clear significant differences between the three treatments (control, DVE, and chitosan NPs loaded with DVE). The average DON concentration decreased after treatment with DVE, and the highest inhibition rate was observed in *F. pseudograminearum* strain 6 (60.23%), while the lowest was recorded for strain 2 (58.35%). When the strains were treated with chitosan NPs loaded with DVE, DON concentrations were further reduced, resulting in a marked increase in the inhibition rate. Under this treatment, the highest inhibition was recorded for *F. pseudograminearum* strain 2 (73.75%), whereas the lowest was observed in strain 6 (68.87%) (Table 5).

It should also be noted that within the same treatment group, the differences between strains were not statistically significant (NS), consistent with the L.S.D. values shown in Table 5.

*F. oxysporum* strain 4, *F. oxysporum* strain 5 and *F. chlamydosporum* strain 8—which are known MON-producing strains—were treated with DVE and NPs loaded with DVE. Statistical analysis confirmed significant differences in MON inhibition among the control, DVE, and NPs loaded with DVE treatments. When free DVE was applied, the highest inhibition rate was observed in *F. oxysporum* strain 4 (53.67%), while the lowest was recorded in *F. oxysporum* strain 5 (48.25%). Upon using NPs loaded with DVE, the inhibition rate increased in all strains, reaching the highest level in *F. chlamydosporum* strain 8 (73.62%), followed by *F. oxysporum* strain 4 (72.09%) and *F. oxysporum* strain 5 (68.18%).

Within each treatment group (control, DVE, and NPs loaded with DVE), there were no statistically significant differences (NS) between MON concentrations of the three *Fusarium* strains. However, numerically, the lowest MON concentration after NPs loaded with DVE treatment was observed in *F. oxysporum* strain 4 (12.44 μg/L), while the highest was recorded in *F. oxysporum* strain 5 (13.25 μg/L) (Table 6).

The presence of *F. pseudograminearum* is considered a strong indicator of potential mycotoxin contamination, particularly deoxynivalenol (DON), as this species is a well-known DON producer. Its detection may reflect a high toxin load in plant tissues, even in the absence of visible disease symptoms [31]. In the present study, our findings agreed with a survey conducted on seventeen wheat fields in the Basra Governorate, where DON was detected in six fields at concentrations ranging from 8 to 1060 µg/kg [21]. HPLC analysis further confirmed the presence of MON toxin, produced by *F. oxysporum* strains 4 and 5, and *F. chlamydosporum* strain 8. To the best of our knowledge, this is the first report to demonstrate MON production by *Fusarium* spp. isolated from wheat samples stored in Iraq. Research on MON remains limited, and further studies are needed to clarify its toxicological impact, behavior under storage and processing conditions, and the efficacy of control strategies [8].

Mycotoxin biosynthesis by *Fusarium* spp. is influenced by multiple factors, including microbial interactions within the matrix, agricultural practices, and environmental conditions such as temperature and humidity. For instance, DON production has been shown to be maximized at approximately 28 °C. Therefore, discrepancies observed among different studies are likely attributable to variations in fungal isolates, substrate composition, and experimental conditions [32].

## 3. Conclusions

In summary, this study demonstrated that *Dodonaea viscosa* extract (DVE) and its nano-formulation effectively inhibited the growth of several *Fusarium* species isolated from stored wheat and significantly reduced the production of the major mycotoxins deoxynivalenol (DON) and moniliformin (MON). The nano-formulated extract exhibited a higher inhibitory effect than the conventional extract, confirming that nanotechnology enhances the antifungal and antimycotoxigenic potential of plant-derived compounds. However, the study was limited to in vitro evaluations and a restricted number of *Fusarium* isolates. Further research should therefore focus on in vivo applications, field-scale validation, and toxicological safety assessments to confirm the practical potential of *Dodonaea viscosa* nanoparticle in grain protection. Additionally, mechanistic studies are needed to elucidate the molecular interactions between the nano-formulated compounds and fungal cell structures. Overall, these findings highlight *Dodonaea viscosa* loaded chitosan nanoparticle as promising eco-friendly alternatives to chemical fungicides for the management of *Fusarium* contamination and mycotoxin accumulation in stored cereals.

## 4. Materials and Methods

### 4.1. Wheat Sampling and Isolation of Fusarium *spp.* Strains from Seeds

During the period from April to May 2021, wheat silos in the eighteen governorates representing the southern, central and northern regions of Iraq: Baghdad (4), Al-Anbar (3), Erbil (3), Babil (3), Basra (2), Dhi Qar (3), Duhok (3), Diyala (2), Karbala (2), Muthanna (2), Nineveh (4), Najaf (3), Qadisiyah (2), Salah al-Din (3), Sulaymaniyah (3), Wasit (4), Ta’mim (3), Maysan (2) were visited for wheat sampling. These regions were characterized by different environmental conditions in terms of temperature, humidity, rainfall and soil type. Samples were collected from the warehouses and silos of the General Company for Grain Trade affiliated with the Iraqi Ministry of Trade. Wheat samples were collected from silos according to the International Standards Organization (ISO) 136,901.2 [33]. The collected material was treated as a composite (bulk) sample, where all subsamples from each batch were combined, homogenized, and subsequently reduced to a 3 kg laboratory sample using a rotary divider.

All samples were placed in labeled plastic bags and stored at 4 °C until analysis. The wheat grains were surface-sterilized using 1% sodium hypochlorite (NaOCl) for 3 min, rinsed twice with sterile distilled water, and then air-dried on sterile filter paper. Ten grains per plate were subsequently cultured on potato dextrose agar (PDA, Merck, Darmstadt, Germany) supplemented with chloramphenicol (50 mg/L) in Petri dishes. The plates were incubated at 25 ± 2 °C for 5 days, after which emerging fungal colonies were subcultured on fresh PDA plates for purification and species identification [34]. Purified isolates were identified at the genus level based on the macroscopic characteristics of the colonies (color, morphology, growth pattern) and microscopic examination using a digital microscope (Olympus, Tokyo, Japan). Microscopic slides were prepared with lactophenol cotton blue stain, and fungal identification was performed according to established taxonomic keys and standard mycological procedures [35].

### 4.2. Identification of Fusarium Strains

**Figure 6:**
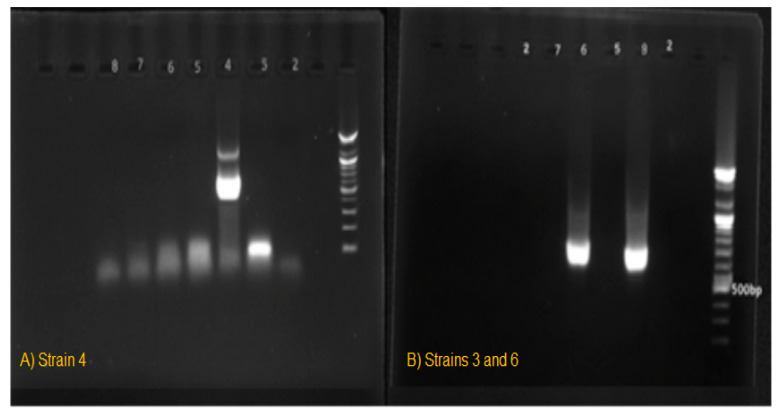
Electrophoresis results of *Fusarium* strains: (**A**) strain 4, (**B**) strain 3 and 6.

The *Fusarium* spp. strains were genetically identified by Polymerase Chain Reaction (PCR) technique using the appropriate primers, ITS1 (5′ TCCTCCGCTTATTGATATGC 3′) and ITS4 (5′ TCCGTAGGTGAACCTGCGG 3′). DNAs of the strains were isolated using the Presto Mini gDNA Yeast kit (GBYB100, Geneaid Biotech, New Taipei City, Taiwan) and PCR was performed in a total volume of 20 μL containing 1 μL of each primer pairs, 6.5 μL AccuPower^®^ PCR PreMix, 5 μL of extracted DNA, and 6.5 μL nuclease free water [36]. Amplification was performed in a thermocycler (Bio-Rad, Pleasanton, CA, USA) under the following conditions: initial denaturation at 95 °C for 5 min, followed by 35 cycles of denaturation at 95 °C for 30 s, annealing at 55 °C for 30 s, and extension at 72 °C for 1 min, with a final extension at 72 °C for 5 min. Prior to sequencing, PCR amplicons were separated by electrophoresis in a 1X TAE Buffer using agarose gel (1% *w*/*v*) stained with ethidium bromide at 0.1 µg/mL. The size of the PCR products was estimated by using a 100 bp size marker (Bioneer, Daejeon, Korea) [37]. Electrophoresis results of *Fusarium* strains (strains 4, 3, and 6) are exhibited in Figure 6.

Commercially prepared PCR amplicons of each strain were sequenced in both forward and reverse directions by an automated DNA sequencer (ABI3730XL) according to the instruction manuals of Macrogen Company (Daejeon, Republic of Korea). The DNA sequences were edited, aligned, and the consensus sequences were generated with BioEdit Sequence Alignment Editor Software version 7.1 (DNASTAR, Madison, WI, USA). Each of the consensus sequences of the *Fusarium* strains was subjected to the Basic Local Alignment Search Tool (BLAST, version 2.15.0+) program and compared with those strains available at the National Center for Biotechnology Information (NCBI) and deposited in GenBank. For phylogenetic analysis, the accession numbers of these strains are listed in Table 1, and MEGAX software (Version 10.1) was used to construct the phylogenetic tree using the neighbor-joining method (Figure 1) [38]. The FASTA sequences of the three *Fusarium* isolates identified in this study were deposited in GenBank (supported by Macrogen, Seoul, Republic of Korea).

### 4.3. Preparation of Alcoholic Extract for Dodonaea viscosa (DVE)

*Dodonaea viscosa* leaves were collected from the gardens of Baghdad University, dried and ground into powder. To prepare the *Dodonaea viscosa extract* (DVE), one liter of ethyl alcohol (96%) was added to 300 g of *Dodonaea viscosa* leaves powder in a glass jar and stirred for three days at room temperature, 28 °C. The mixture was filtered with a Buchner funnel and then filtered with a glass funnel to remove impurities. A rotary evaporator was used to remove the solvent and dry the solution at 40–60 °C. After three hours, a viscous liquid was obtained and placed in tightly sealed containers in the freezer at −20 °C until used in the experiment [39]. After solvent evaporation, a viscous crude extract was obtained (48.6 g from 300 g of dry leaf powder), corresponding to an extraction yield of 16.2% (*w*/*w*).

### 4.4. Identification of the Major Components of DVE Using GC-MS System

A GC-MS system QP2010 model (Shimadzu, Kyoto, Japan) was used for the identification of components of DVE. Helium was used as a carrier gas, and a micro-syringe was used to inject eight µL of DVE into the GC-MS system. The column temperature program started at 40 °C. The injector temperature was 280 °C while the detector was at 200 °C. The split ratio was 1:30. The data was processed using software to match and identify the organic compounds in the extracts. The results were evaluated by comparing the retention indices and fragmentation patterns of the mass spectra with those stored in a computer library. The compounds were identified by comparing their spectra with those in the NIST08.LIB mass spectra libraries [40].

### 4.5. Biosynthesis of Chitosan Nanoparticles Loaded DVE

Chitosan (AC00671K, Avonchem Ltd., Macclesfield, UK) was purchased commercially. It was dissolved in acetic acid (200 mg/100 mL acetic acid) and mixed with *Dodonaea viscosa* extract (1 g DVE/100 mL DW) in equal amounts, and the reaction was carried out using a reflux distillation apparatus. The product was separated using syringe filters, washed three times with pure methanol, hot distilled water to remove polar and irrelevant compounds, and finally dried in an electric oven at 50 °C [41].

The ion coagulation method was used to produce chitosan nanoparticles loaded with DVE. Acetic acid was used as a solvent at 1%, and 5 mg/mL of the product (chitosan NPs loaded with DVE) was dissolved in it. Tripolyphosphate (TPP) was used at a 1:2.5 (*w*/*w*) ratio and stirring continued while adjusting the pH to 7 until a clear solution was obtained. This reaction was carried out at 25 °C and continued for 6 h [16].

### 4.6. Measurement and Morphological and Structural Characterization of Nanoparticles

A dual-beam UV–Vis spectrophotometer (Shimadzu UV-1800, Kyoto, Japan) was used, and all spectra were recorded using a quartz cuvette with a 1 cm optical path length at room temperature. Distilled deionized water was used as the blank. Absorbance was measured in the range of 200–800 nm, and the concentrated samples were diluted 1:10 prior to analysis [42]. Fourier-transform infrared (FT-IR) spectroscopy was performed to detect the vibrational transitions of the functional groups, which occur in the mid-infrared region (4000–400 cm^−1^). The spectra were recorded using an FT-IR spectrometer (Shimadzu 8400S, Japan), and the characteristic absorption bands were analyzed to identify the functional groups present in the sample [43]. The surface morphology of nanoparticles was examined by atomic force microscopy (AFM, NaioAFM2022, Nanosurf AG, Switzerland), where samples to be examined were dropped and distributed on the microscope platform, and the analysis was carried out according to the procedure followed by [44]. A Scanning Electron Microscope (SEM, TESCAN-MIRA3 Czech Republic) was used to investigate the morphology of chitosan NPs-loaded DVE at different magnifications (100 kX, 50 kX, and 30 kX) [45].

### 4.7. In Vitro Efficacy of DVE and Chitosan NPs Loaded with DVE in Inhibiting the Growth of Fusarium *spp.*

The DVE was added at three concentrations (0.5, 1, and 1.5%) to each 100 mL of PDA after autoclave. After the medium solidified, all plates were inoculated with each freshly grown *Fusarium* spp. strains (1 cm piece in size taken with a cork hole) and placed in the center of the plate. The control group consisted of Petri dishes containing standard PDA (potato dextrose agar) medium (without added DVE/nanoparticles), inoculated with *Fusarium* strains tested only. The experiment was conducted at a rate of three replicates for each concentration and incubated at 25 °C for seven days in an incubator. The same previous steps were carried out to examine the effect of the DVE-loaded chitosan NPs with the concentration of 0.75% being used, (representing the average concentrations used as extract) [46]. The inhibition rate was calculated using follower formula [47].

Inhibition percentage (%) = ((R − R1)/R) × 100(1)

where

R = growth diameter rate in control dishes (distance measured in mm);

R1 = growth diameter rate in treatment dishes.

### 4.8. Testing the DON and MON Mycotoxins Production from Fusarium *spp.* Strains After DVE and Chitosan NPs Loaded with DVE Treatments

For mycotoxin induction, 150 g of wheat was moistened with 100 mL of distilled water to serve as the culture substrate. The glass incubation bottles were sterilized in an autoclave at 121 °C and 1.5 kg/cm^2^ pressure for 20 min, after which they were inoculated with *Fusarium* spp. previously grown on PDA. The inoculated wheat was thoroughly mixed and incubated at 25 ± 2 °C for 21 days. The DVE and its nano-chitosan-loaded formulation were applied only during the active toxin production phase at different concentrations. The reduction in DON and MON levels as a direct result of the extract/nanoparticle interference with active mycotoxin biosynthesis was quantified using high-performance liquid chromatography (HPLC). A HPLC system (Syknm S4011, Stuttgart, Germany) was used to measure DON and MON concentrations, following previously reported protocols with minor modifications [48,49].

For the extraction of DON and MON, 20 g of sample was mixed with 100 mL of distilled water in Nalgene bottles and shaken for 1 h using a reciprocating shaker. The mixture was then centrifuged at 3200× *g* for 5 min and filtered through Whatman No. 1 folded filter paper. A 2 mL aliquot of the filtrate was diluted to 10 mL with distilled water and passed through a 0.45 µm membrane filter using a micro-syringe for clarification. For HPLC confirmation, another 2 mL aliquot was diluted to 10 mL with water and loaded onto an Oasis HLB cartridge preconditioned with 5 mL of methanol followed by 5 mL of water. The cartridge was washed with 2 mL of water, dried under vacuum for 15 min, and eluted with 4 mL of methanol. The eluate was evaporated to dryness under a gentle nitrogen stream and reconstituted in 200 µL of methanol/0.1% aqueous acetic acid + 1 mM ammonium acetate (20:80, *v*/*v*). HPLC separation was performed using a Zorbax SB-C18 column (25 cm × 4.6 mm i.d.) with a mobile phase consisting of water and acetonitrile (30:70, *v*/*v*) at a flow rate of 1.2 mL/min [50]. Calibration curves were constructed using certified DON and MON standards within the range of 1–5 µg/L. The calibration curves showed linearity with R^2^ > 0.999 for both toxins. The retention time was 3.9 and 6 min for deoxynivalenol and moniliformin, respectively. LOD and LOQ values were 0.062 and 0.189 µg/L for moniliformin while they were 0.137 and 0.415 µg/L for deoxynivalenol, respectively. The following equation was used to calculate the mycotoxin inhibition ratio:

Inhibition ratio (%) = ((B0 − B1)/B0) × 100(2)

B0, B1 values represent the DON and MON mycotoxin concentration produced before treatment (control group), and after treatment, respectively [51]. (Figures S1–S4 shows the chromatograms of mycotoxin standards, before and after treatments).

### 4.9. Statistical Analysis

Laboratory experiments were conducted using three replicates for each treatment [52]. The Statistical Analysis System-SAS (Version 9.6) program was used to detect the effect of different factors on study parameters. This study used the least significant difference (LSD) to significantly compare means (ANOVA/One-way).

## Figures and Tables

**Table 1:** The results of PCR analysis of seven *Fusarium* strains.

Sample	Identity of the Strains	Location	GenBank acc. No
2	*Fusarium pseudograminearum*	Iraq	PQ721291.1
3	*Fusarium pseudograminearum*	Iraq	MT465500.1
4	*Fusarium oxysporum*	Iraq	PQ721292.1
5	*Fusarium oxysporum*	Pakistan	MN272279.1
6	*Fusarium pseudograminearum*	Iraq	MT465500.1
7	*Fusarium pseudograminearum*	China	MZ312460.1
8	*Fusarium chlamydosporum*	Iraq	PQ721294.1

**Table 2:** Mean inhibition percentage (%) of *Fusarium* strains treated with different concentrations of *Dodonaea viscosa* extract (DVE).

Strains	Inhibition Ratio (%) After 0.5% DVE Treatment	Inhibition Ratio (%) After 1% DVE Treatment	Inhibition Ratio (%) After 1.5% DVE Treatment	L.S.D.
*F. pseudograminearum* strain 2	61.11 ^Ab^	66.25 ^Aab^	68.00 ^Aa^	6.092 *
*F. pseudograminearum* strain 3	34.25 ^Ca^	36.75 ^Ca^	39.25 ^Ca^	5.188 NS
*F. oxysporum* strain 4	35.85 ^Cb^	39.02 ^Cab^	42.43 ^Ca^	5.863 *
*F. oxysporum* strain 5	63.37 ^Ab^	67.13 ^Aab^	68.94 ^Aa^	5.337 *
*F. pseudograminearum* strain 6	15.16 ^Db^	20.64 ^Dab^	22.58 ^Da^	5.074 *
*F. pseudograminearum* strain 7	58.23 ^Ab^	63.62 ^Aab^	64.76 ^Aa^	5.859 *
*F. chlamydosporum* strain 8	45.00 ^Bb^	51.75 ^Ba^	55.00 ^Ba^	6.045 *
L.S.D.	7.537 *	7.911 *	6.805 *	---

Inhibition (%) was calculated using the formula [(R − R1)/R] × 100, where R = growth diameter in control and R1 = growth diameter in treated plates. Different superscript letters (a–b within rows, A–D within columns) indicate statistically significant differences based on LSD (Least Significant Difference) test at *p* < 0.05. * shows statistically significant differences. NS means no significant difference.

**Table 3:** Identified major components of ethanolic leaf extract of *Dodonaea viscosa* using GC-MS.

Peak	Retention Time (min)	Area (%)	Name
1	13.612	0.6533	Catechol
2	18.309	0.437	Methyl 4-O-methyl-.beta.-D-xylopyranoside
3	20.183	0.537	Benzene, 1,2-dimethoxy-4-(1-propenyl)-
4	21.588	0.5637	Neophytadiene
5	22.858	0.6602	n-Hexadecanoic acid
6	23.197	0.3448	2H-2,4a-Ethanonaphthalene, 1,3,4,5,6,7-hexahydro-2,5,5-trimethyl-
7	24.019	2.509	Diallyl phenyl vinylsilane
8	24.548	0.7617	9,12,15-Octadecatrienoic acid, (Z,Z,Z)-
9	27.067	3.2703	Phosphinic acid, diphenyl-, methyl ester
10	27.725	1.5871	4H-1-Benzopyran-4-one, 2,3-dihydro-5,7-dihydroxy-2-phenyl-, (S)-
11	27.814	3.0364	Alizarin, 1,2-O-(phenylboranediyl)-
12	28.323	9.2764	3-Heptanol, TMS derivative
13	28.526	6.6795	(3aR,9aS)-2,2,3a-Trimethyl-3,3a,4,5-tetrahydroindeno [7a,1-b]furan-7(2H)-one
14	29.158	32.2167	4-Cyclohexyl-1-(furan-2-ylmethyl)-4H,5H,7H-pyrazolo [3,4-b]pyridin-6-one
15	29.484	9.379	6-Methoxy-2-[2-[imidazol-1-yl]ethoxy]-8-nitroquinoline
16	29.585	2.919	Cyclohexanol, 1-ethynyl-
17	29.959	0.9615	16-Heptadecyn-4-one, 1,2-dihydroxy-
18	30.549	0.4411	Eicosane
19	30.773	1.169	trans-1-Methyl-7-methylenebicyclo(4.4.0)decan-3-one
20	31.554	1.9718	2-(4-Hydroxyphenyl)-3,6,7-trimethoxy-5-hydroxy-4H-1-benzopyran-4-one (penduletin)
21	32.118	0.5439	Eicosane
22	32.375	9.8093	4′,5,7-Trihydroxy-3,6-dimethoxyflavone
23	32.437	1.1655	Vitamin E
24	32.586	0.9355	Naphthalene, 6-(1-ethylpropyl)-1,2,3,4-tetrahydro-
25	32.742	1.6076	3,4′-Di-O-methyleupalitin
26	33.665	3.8315	2-(4-Hydroxyphenyl)-3,6,7-trimethoxy-5-hydroxy-4H-1-benzopyran-4-one (penduletin)
27	36.211	2.7323	D:A-Friedooleanane

**Table 4:** Mean inhibition percentage (%) of *Fusarium* strains treated with the chitosan NPs loaded with DVE.

Strains	Inhibition Ratio (%) After Chitosan NPs Loaded with DVE
*F. pseudograminearum* strain 2	64.70 ^ABC^
*F. pseudograminearum* strain 3	67.05 ^ABC^
*F. oxysporum* strain 4	65.88 ^ABC^
*F. oxysporum* strain 5	63.52 ^BC^
*F. pseudograminearum* strain 6	68.23 ^AB^
*F. pseudograminearum* strain 7	70.58 ^A^
*F. chlamydosporum* strain 8	61.17 ^C^
L.S.D. value	6.429 *

The presence of different capital letters in the same column represents the values of the effect of the chitosan NPs-loaded DVE at a certain concentration on all strains. * shows statistically significant differences

**Table 5:** Deoxynivalenol (µg/L) concentrations of *Fusarium* strains after treatment with the DVE and chitosan NPs loaded with DVE and inhibition ratios.

Strains	DON Before Treatment (µg/L)	DON After DVE Treatment (µg/L)	DON After Chitosan NPs Loaded with DVE (µg/L)	Inhibition Ratio % After DVE Treatment	Inhibition Ratio % After Chitosan NPs Loaded with DVE
*F. pseudograminearum* strain 2	43.82 ± 2.17	18.25 ± 1.05	11.50 ± 0.74	58.35	73.75 ^A^
*F. pseudograminearum* strain 3	47.24 ± 3.26	19.11 ± 1.48	12.65 ± 0.91	59.67	73.22 ^A^
*F. pseudograminearum* strain 6	43.86 ± 2.37	17.44 ± 0.97	13.65 ± 0.96	60.23	68.87 ^B^
*F. pseudograminearum* strain 7	47.23 ± 3.61	18.95 ± 1.29	12.65 ± 0.88	59.87	73.21 ^A^
L.S.D. value	3.551 NS	1.802 NS	2.076 NS	5.911 NS	3.967 *

Means having different capital letters in the same column differed significantly, (*p* < 0.05). NS means no significant difference. * shows statistically significant differences.

**Table 6:** Moniliformin (µg/L) concentrations of *Fusarium* strains after treatment with the DVE and chitosan NPs loaded with DVE and inhibition ratios.

Strains	MON Before Treatment (µg/L)	MON After DVE Treatment (µg/L)	MON After Chitosan NPs Loaded with DVE (µg/L)	Inhibition Ratio % after DVE Treatment	Inhibition Ratio % After Chitosan NPs Loaded with DVE
*F. oxysporum* strain 4	44.58 ± 2.79	20.65 ± 1.63	12.44 ± 0.74	53.67 ^A^	72.09 ^AB^
*F. oxysporum* strain 5	41.65 ± 2.05	21.55 ± 1.48	13.25 ± 0.93	48.25 ^B^	68.18 ^B^
*F. chlamydosporum* strain 8	42.65 ± 3.18	19.80 ± 1.04	11.25 ± 0.62	53.57 ^A^	73.62 ^A^
L.S.D. value	3.094 NS	2.067 NS	2.178 NS	3.962 *	4.208 *

Means having with different capital letters in same column differed significantly, * (*p* ≤ 0.05). * shows statistically significant differences. NS means no significant difference.

## Data Availability

The original contributions presented in this study are included in the article. Further inquiries can be directed to the corresponding authors.

## Supplementary Materials

The following supporting information can be downloaded at: https://www.mdpi.com/article/10.3390/toxins17110551/s1, Figure S1. Deoxynivalenol and Moniliformin standard chromatogram by HPLC chromatographic analysis. (A) DON standard results, (B) MON standard. Figure S2. Chromatograms of deoxynivalenol and moniliformin before treatment by HPLC chromatography analysis; (sample 2) *F. pseudograminearum* strain 2, (sample 3) *F. pseudograminearum* strain 3, (sample 4) *F. oxysporum* strain 4, (sample 5) *F. oxysporum* strain 5, (sample 6) *F. pseudograminearum* strain 6, (sample 7) *F. pseudograminearum* strain 7, (sample 8) *F. chlamydosporum* strain 8. Figure S3. Chromatograms of deoxynivalenol and moniliformin after treatment of *Dodoneae viscosa* extract (DVE) by HPLC chromatography analysis (sample 2) *F. pseudograminearum* strain 2, (sample 3) *F. pseudograminearum* strain 3, (sample 4) *F. oxysporum* strain 4, (sample 5) *F. oxysporum* strain 5, (sample 6) *F. pseudograminearum* strain 6, (sample 7) *F. pseudograminearum* strain 7, (sample 8) *F. chlamydosporum* strain 8. Figure S4. Chromatograms of deoxynivalenol and moniliformin after treatment of chitosan NPs loaded DVE by HPLC chromatography analysis (sample 2) *F. pseudograminearum* strain 2, (sample 3) *F. pseudograminearum* strain 3, (sample 4) *F. oxysporum* strain 4, (sample 5) *F. oxysporum* strain 5, (sample 6) *F. pseudograminearum* strain 6, (sample 7) *F. pseudograminearum* strain 7, (sample 8) *F. chlamydosporum* strain 8.
