# Supplementary material for: Efficacy of Dodonaea viscosa Extract and Its Chitosan-Based Nanoparticle Formulation on the Growth of Fusarium Strains and the Production of Deoxynivalenol and Moniliformin in Stored Wheat
Source: Toxins (Basel). 2025 Nov 5;17(11):551. doi: 10.3390/toxins17110551 (PMC12656638; doi:10.3390/toxins17110551)
Supplement: Supplementary file 1 [file toxins-17-00551-s001.zip › toxins-3912410-supplementary.pdf]

Article

Efficacy of *Dodonaea viscosa* Extract and Its Chitosan-Based Nanoparticle Formulation on the Growth of *Fusarium* Strains and the Production of Deoxynivalenol and Moniliformin in Stored Wheat

Hussein Ali Salman Alsahho [1,2,*], Sumer Horuz [3,*] and Kevser Karaman [4]

[1] Graduate School of Natural and Applied Sciences, Erciyes University, 38280, Kayseri, Türkiye
[2] Iraqi Ministry of Trade-Grain Board of Iraq, Bab-Al Moazzam, Baghdad 329, Iraq
[3] Faculty of Agriculture, Department of Plant Protection, Erciyes University, 38280 Kayseri, Türkiye
[4] Faculty of Agriculture, Department of Agricultural Biotechnology, Erciyes University, 38280 Kayseri, Türkiye; kevserkaraman@erciyes.edu.tr (K.K)
* Correspondence: hussienalsa327@gmail.com (H.A.A.-S.); shoruz@erciyes.edu.tr (S.H.)

1. Supplementary

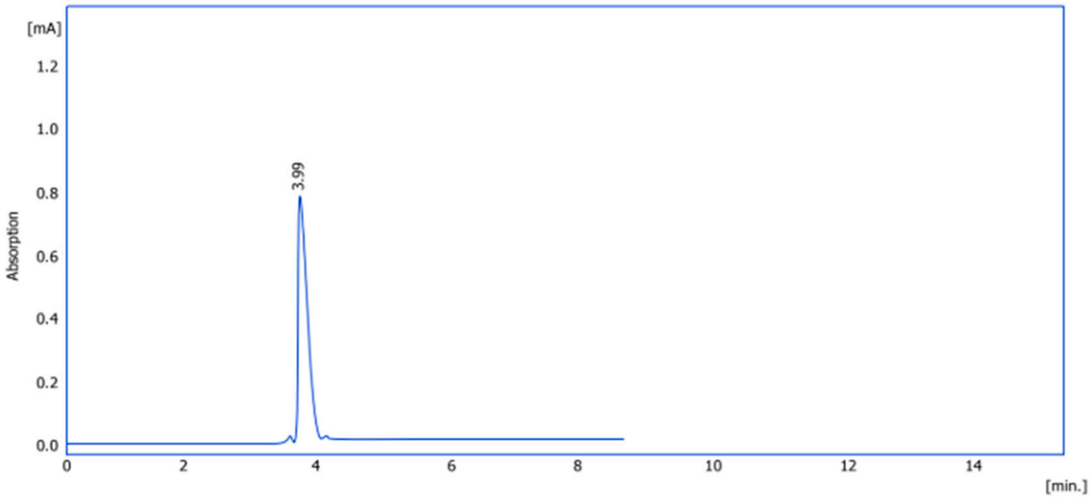

Result chromatography Table (Uncal - F:\ Deoxynivalenol 2 ppb)

No	Reten. Time [min]	Area [mAU.s]	Height [mAU]	Area [%]	Height [%]	W05 [min]	Compound Name
1	3.99	1425.99	794.08	100.00	100.00	0.25	
	Total	1425.99	794.08	100.00	100.00		

A

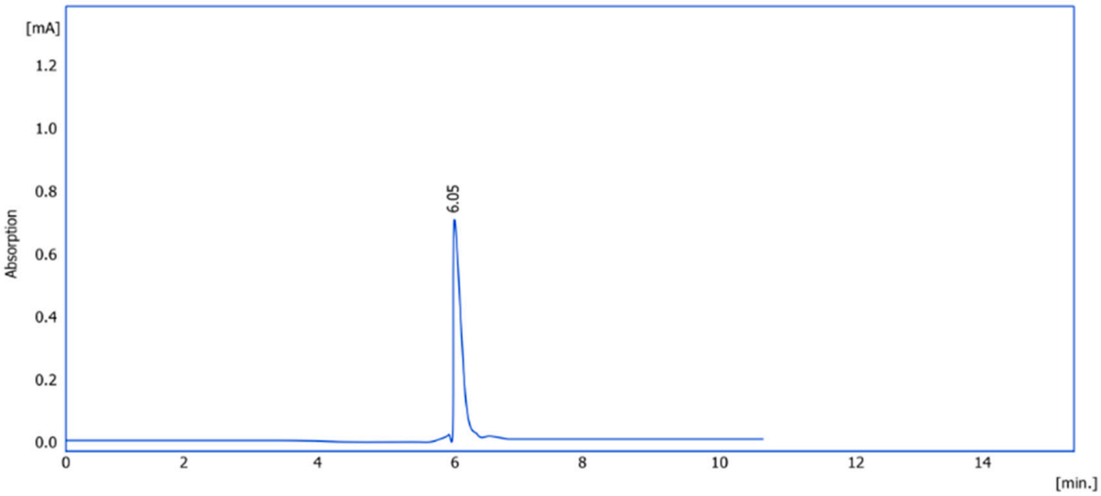

Result chromatography Table (Uncal - F:\ Moniliformin 2 ppb)

No	Reten. Time [min]	Area [mAU.s]	Height [mAU]	Area [%]	Height [%]	W05 [min]	Compound Name
1	6.05	1745.14	784.00	100.00	100.00	0.25	
	Total	1745.14	784.00	100.00	100.00		

B

Figure S1. Deoxynivalenol and Moniliformin standard chromatogram by HPLC chromatographic analysis. (A) DON standard results, (B) MON standard.

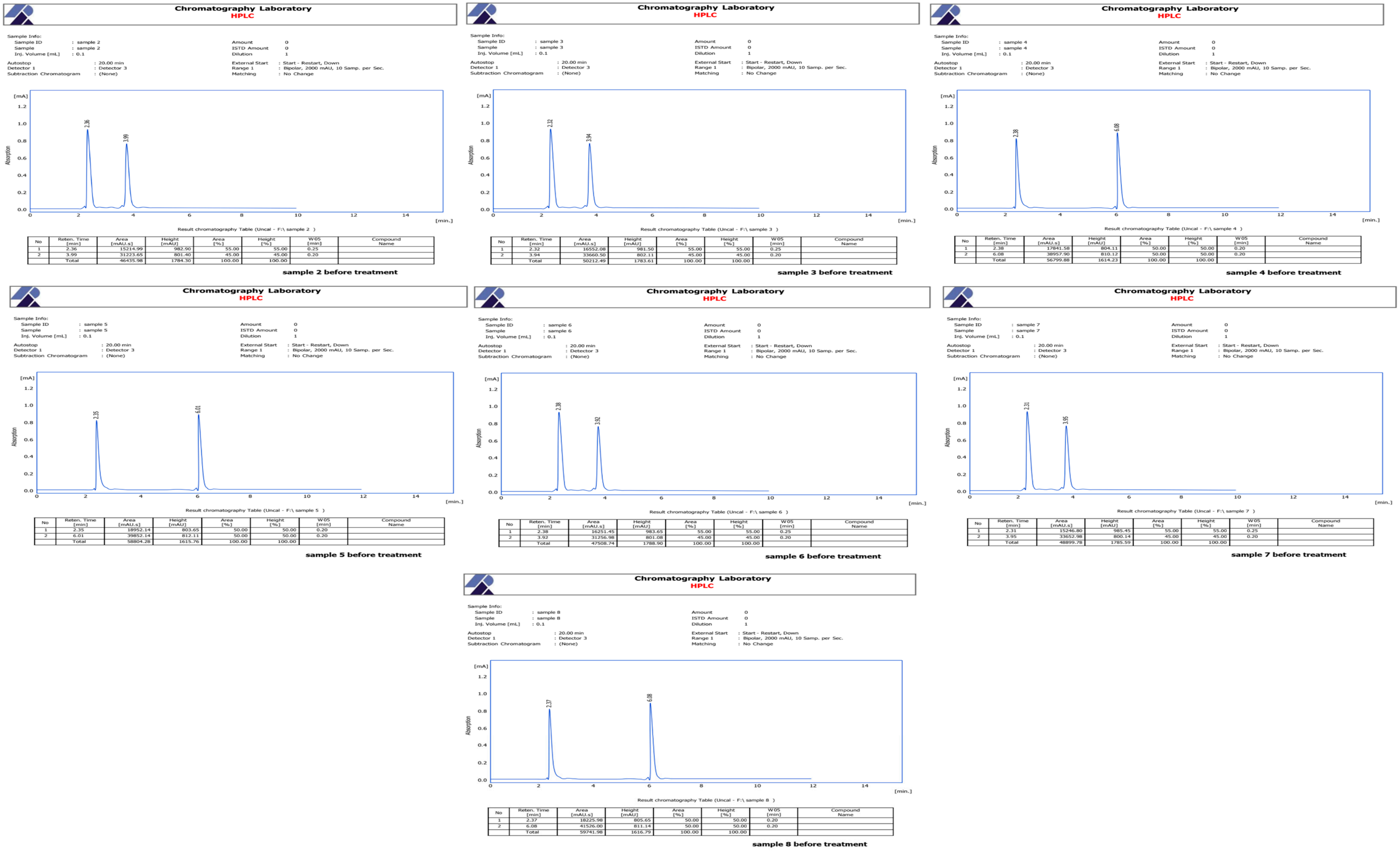

Figure S2. Chromatograms of deoxynivalenol and moniliformin before treatment by HPLC chromatography analysis. (sample 2) *F. pseudograminearum* strain 2, (sample 3) *F. pseudograminearum* strain 3, (sample 4) *F. oxysporum* strain 4, (sample 5) *F. oxysporum* strain 5, (sample 6) *F. pseudograminearum* strain 6, (sample 7) *F. pseudograminearum* strain 7, (sample 8) *F. chlamydosporum* strain 8.

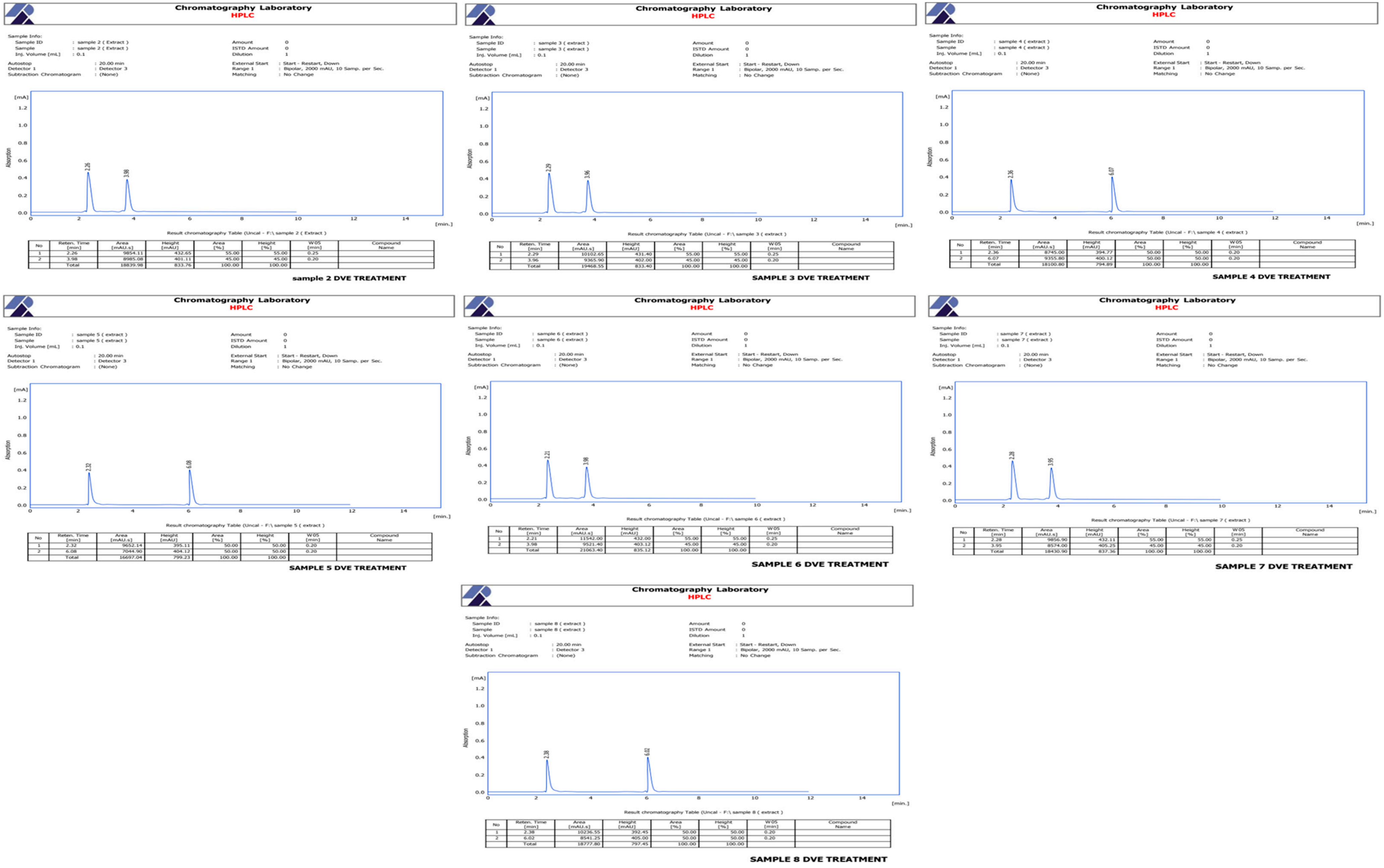

Figure S3. Chromatograms of deoxynivalenol and moniliformin after treatment of *Dodoneae viscosa* extract (DVE) by HPLC chromatography analysis (sample 2) *F. pseudograminearum* strain 2, (sample 3) *F. pseudograminearum* strain 3, (sample 4) *F. oxysporum* strain 4, (sample 5) *F. oxysporum* strain 5, (sample 6) *F. pseudograminearum* strain 6, (sample 7) *F. pseudograminearum* strain 7, (sample 8) *F. chlamydosporum* strain 8.

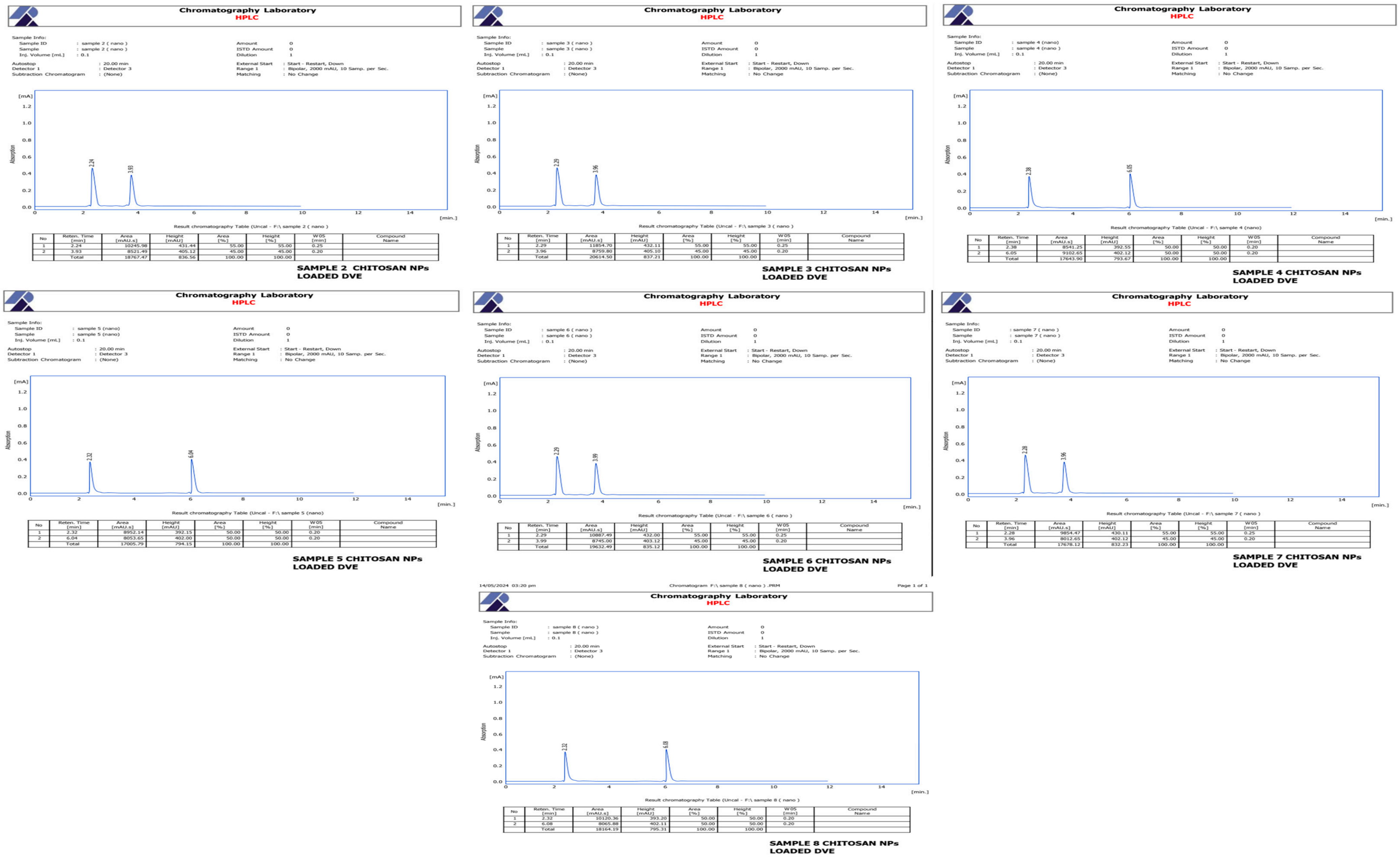

Figure S4. Chromatograms of deoxynivalenol and moniliformin after treatment of chitosan NPs loaded DVE by HPLC chromatography analysis (sample 2) *F. pseudograminearum* strain 2, (sample 3) *F. pseudograminearum* strain 3, (sample 4) *F. oxysporum* strain 4, (sample 5) *F. oxysporum* strain 5, (sample 6) *F. pseudograminearum* strain 6, (sample 7) *F. pseudograminearum* strain 7, (sample 8) *F. chlamydosporum* strain 8.
